# Analytical and functional approaches to assess the immunogenicity of gluten proteins

**DOI:** 10.3389/fnut.2022.1049623

**Published:** 2023-01-18

**Authors:** Gianfranco Mamone, Luigia Di Stasio, Serena Vitale, Stefania Picascia, Carmen Gianfrani

**Affiliations:** ^1^Institute of Food Science, Department of Biology, Agriculture and Food Sciences, National Research Council of Italy, Avellino, Italy; ^2^Institute of Biochemistry and Cell Biology, Department of Biomedical Sciences, National Research Council of Italy, Naples, Italy

**Keywords:** gluten immunogenic peptides (GIP), INFOGEST digestion method, gastrointestinal proteases, T-cell assays, short gluten challenge, celiac disease

## Abstract

Gluten proteins are the causative agents of celiac disease (CD), a lifelong and worldwide spread food intolerance, characterized by an autoimmune enteropathy. Gluten is a complex mixture of high homologous water-insoluble proteins, characterized by a high content of glutamine and proline amino acids that confers a marked resistance to degradation by gastrointestinal proteases. As a consequence of that, large peptides are released in the gut lumen with the potential to activate inflammatory T cells, in CD predisposed individuals. To date, several strategies aimed to detoxify gluten proteins or to develop immunomodulatory drugs to recover immune tolerance to gluten are under investigation. This review overviews the state of art of both analytical and functional methods currently used to assess the immunogenicity potential of gluten proteins from different cereal sources, including native raw seed flours and complex food products, as well as drug-treated samples. The analytical design to assess the content and profile of gluten immunogenic peptides, described herein, is based on the oral-gastro-intestinal digestion (INFOGEST model) followed by extensive characterization of residual gluten peptides by proteomic and immunochemical analyses. These approaches include liquid chromatography–high-resolution mass spectrometry (LC-MS/MS) and R5/G12 competitive ELISA. Functional studies to assess the immune stimulatory capabilities of digested gluten peptides are based on gut mucosa T cells or peripheral blood cells obtained from CD volunteers after a short oral gluten challenge.

## 1. Introduction

### 1.1. Epidemiology, diagnosis, and therapy

Celiac disease (CD) is a common immune-mediated enteropathy caused in genetically susceptible individuals by the consumption of gluten proteins, contained in wheat, barley, and rye cereals (1). The genetic predisposition is given by specific alleles of the HLA class II DQ genes that encode for the DQ2.5 (DQA1*05 and DQB1*02) or DQ8 (DQA1*03 and DQB1*0302) molecules (2). CD can arise at any age, with a global prevalence ranging from 0.5 to 2%, with an average of 1% in wheat consuming countries (1).

Clinically, CD may manifest with gastrointestinal symptoms, such as abdominal pains, diarrhea, anemia, and malabsorption syndromes (typical CD forms), or may present with extra-intestinal symptoms, such as skin lesions, ataxia, infertility, and frequent aphthous ulcerations (atypical CD forms). However, it may manifest symptomless, despite an evident intestinal mucosa inflammation (silent CD forms) (3). Diagnostic criteria include serological tests, such as the detection of anti-tissue transglutaminase antibodies (tTG-IgA) and anti-endomysium (EMA-IgA). Anti-deamidated gliadin peptides (DGP-IgG) are also included but have a specificity lower than tTG-IgA (average value: 97.9 and 87.81% for tTG and DPG, respectively) (4). Notwithstanding the high sensitivity and specificity of these serological tests, histological evaluation of duodenal mucosa after an esophagogastroduodenoscopy (EGD) is still considered the gold standard to make a diagnosis of CD, in particular in adults (5). Instead, in childhood, less invasive criteria are recommended according to recent guidelines of the European Society for Pediatric Gastroenterology Hepatology and Nutrition (ESPGHAN). A diagnosis of CD can be performed in the presence of high titers of tTG-IgA (at least 10-fold the cutoff), and the presence of clear CD-associated symptoms, thus no more requiring the EGD (6).

Furthermore, approximately 10% of children with positive tTG-IgA show duodenal mucosa with normal villous architecture and low inflammation. These patients are considered to have a “potential” CD. Notably, most of the subjects included in this group are at high risk for developing a typical CD later in life (7, 8).

To date, no drug is available for celiac disease patients, and the only safe and efficient treatment is the gluten-free diet (GFD) which is to be strictly followed for life. Gluten removal from the diet results in symptom recovery and small intestinal lesion resolution in the great majority of dietary compliant patients (9, 10).

### 1.2. Genomic and chemistry of gluten proteins

Wheat is one of the world’s major cultivated and consumed food crops along with rice and maize.^1^ It is an essential staple food because of its important nutritional characteristics, technological properties, and long shelf life (11). Bread wheat (*Triticum aestivum*) and durum wheat (*Triticum durum*) are the most important varieties currently grown worldwide. Bread wheat accounts for 95% of global wheat production and is used for the manufacturing of bakery foods (i.e., bread, cakes, and cookies). Durum wheat is primarily used for the production of pasta. The modern cultivated wheat has passed a long evolution that took place for more than 10,000 years starting with polyploidization events between *Triticum urartu* (AA genome) and an *Aegilops speltoides*-related species (BB genome) and resulting in *Triticum turgidum ssp. dicoccoides*, and between *T. turgidum ssp. durum* (AABB genome) and *Aegilops tauschii* (DD genome), forming the modern hexaploid bread wheat (AABBDD genome) (12). Historically, the large diffusion of hexaploid and tetraploid wheat cultivars was due to their adaptability and high yield potential, as well as to the capacity of gluten proteins to confer viscoelastic properties that allow the dough to be processed into food products (13, 14).

Gluten proteins account for about 85–90% of wheat protein fraction, while the remainder is constituted by the water-soluble albumin and globulin proteins. According to Osborne’s classification, gluten proteins are divided into alcohol-soluble monomeric gliadin and alcohol-insoluble polymeric glutenin. The gliadins are further classified into α-, γ-, and ω-gliadin fractions with molecular mass ranging from 28 to 55 kDa. Gliadins are important in determining the extensibility characteristics of dough. During the mixing of flour with water, gliadins take part in the development of the gluten network through the formation of intermolecular hydrogen bonds and hydrophobic bonds between non-polar amino acid side chains, which also interact with the flour lipids (15–17).

Glutenins consist of disulfide-linked proteins with molecular mass ranging from approximately 60,000 to more than 10 million. Following the reduction of inter-chain and intra-chain disulfide bonds, glutenins are divided into high molecular weight (70–90 kDa) glutenin subunits (HMW-GS) and low molecular weight (20–45 kDa) glutenin subunits (LMW-GS), which represent the 40 and 60% of glutenin composition, respectively (14). Structurally, HMW-GS consists of three domains characterized by a non-repetitive N-terminal domain and a C-terminal domain, which contain cysteine residues, that produce inter-chain and intra-chain disulfide bonds, and a repetitive central domain that promotes intermolecular hydrogen bonding (18). The number of cysteine residues of HMW-GS affects the rheological properties of the dough, since disulfide bonds determine gluten extensibility and elasticity through the formation of larger glutenin aggregates (19).

### 1.3. Proteolytic resistance of gluten proteins and role in CD immunopathogenesis

Gluten proteins are poorly affected by proteases occurring in the gastric and intestinal tract (GI), including the enzymes of the small intestinal brush-border membrane. The marked resistance to proteolysis is due to the high percentage of proline and glutamine residues and to the lack of specific GI proteases with cleavage-site activity for intra-chain post-proline residues (20). As a consequence, large gluten oligopeptides reach the gut lumen, triggering adverse immune responses in patients with CD (21). To date, several gluten peptides resistant to digestive enzymes and harboring immune toxic sequences have been identified (22, 23). Two peptides derived from α- and γ-gliadin are particularly relevant for the activation of intestinal T cells: the 33-mer (LQLQPFPQPQLPYPQPQLPYPQPQLPYPQPQPF) from α-2-gliadins and the 26-mer (FLQPQQPFPQQPQQPYPQQPQQPFPQ) from γ-gliadins. These peptides contain shorter sequences that, after TG2-mediated deamination, bind HLA-DQ2 and HLA-DQ8 molecules and stimulate T cells in patients with CD (11, 23–25).

Among protease-resistant sequences, peptide 31–55 from α-gliadins (LGQQQPFPPQQPYPQPQPFPSQQPY) has been found to contribute to the onset and the development of CD (26, 27). Its shorter peptide, 31–43 (LGQQQPFPPQQPY) elicits an innate immune response in professional antigen presenting cells (monocytes, macrophages, and dendritic cells) and expression of stress signals on intestinal epithelial cells (28–30).

Several studies have demonstrated that intestinal CD4 + T cells have a key role in inflammatory responses in CD (25, 31–33), and that all gluten protein families contain peptides able to stimulate T-cell response (23). The activation of these T cells, resident mainly in the lamina propria compartment, triggers an inflammatory cascade mediated by interferon-γ (INF-γ) and interleukin-21 (IL-21). These gluten-specific CD4 + T cells can be isolated from CD intestinal mucosa and *in vitro* expanded, thus representing an important source for bioassays useful to assess pathogenesis and to validate novel therapies for CD management, as reported below in more detail.

## 2. Analytical assessment of gluten peptides immunotoxicity

### 2.1. *In vitro* enzymatic digestion of gluten proteins

Understanding the outcome of the gluten proteins in the human digestive system (bioavailability) is an area of interest being these dietary proteins are the causative factor of CD.

Food digestion may be assessed by both *in vivo* (human or animal) and *in vitro* methods, although the latter procedures are preferred in research related to food and nutrition because of their speed, cost, and reproducibility compared to *in vivo* studies (34). Models of *in vitro* digestion have been proposed since the 1990s to study the metabolism of food components along with the gastrointestinal tract. Regarding the CD studies, early *in vitro* digestion experiments were limited to pepsin and trypsin proteolysis for mimicking the gastric and pancreatic stages, respectively (25). Shan et al. (35) used a more accurate *in vitro* digestion consisting of a pool of duodenal enzymes (trypsin, chymotrypsin, elastase, and carboxypeptidase) along with brush-border membrane enzymes to mimic the duodenal and intestinal digestion, respectively. With this strategy, the authors identify the well-known 33-mer from α-gliadin and 26-mer from γ-gliadin. A separate study employed the *in vitro* methods to demonstrate the digestive protease resistance of a 25-mer from γ-gliadin (26). Similarly, Gianfrani et al. assessed the immunogenicity of diploid wheat *Triticum monococcum* after *in vitro* digestion (22). The authors found that monococcum gluten proteins are more susceptible to the digestion of GI proteases compared to those from hexaploidy wheat leading to an overall lower immunotoxicity on T cells (22).

The *in vitro* digestive models have been improved extensively over the years. Noteworthy, an *in vitro* model has been successfully designed to reproduce the physiological process as close as possible to the *in vivo* one (36, 37). The INFOGEST digestion procedure consists of three consecutive phases: oral, gastric, and intestinal (small intestine), which include all enzymes, bile salts, digestive fluids (saliva, gastric, and intestinal juices), and incubation times of physiological condition. Following a pilot publication in 2016, the INFOGEST model is the most used *in vitro* digestion method throughout the research community aimed to simulate the behavior of foods within the human gastrointestinal tract (38). The INFOGEST method was also applied to the study of the immunogenic potential of peptides from wheat products, tree nuts, and peanuts, which are all resistant to gastrointestinal digestion (39–42).

### 2.2. Detection of gluten peptides after the *in vitro* gastroduodenal digestion

**FIGURE 1:**
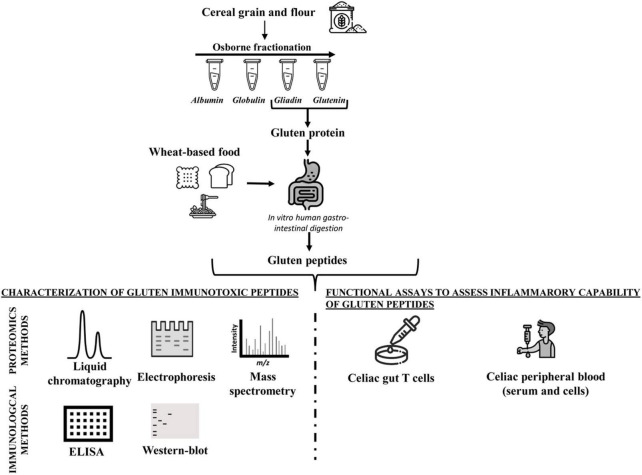
Workflow of analytical and functional approaches to assess the amount and immunogenicity of gluten proteins in raw seed flour and complex food matrices.

Because of the physicochemical properties of gluten proteins, there are, to date, few approaches validated to detect gluten toxic peptides present in different food matrices. In general, the main analytical methods employed to study the gluten protein are electrophoresis (43–46), reversed-phase (RP)-HPLC, size-exclusion HPLC (SE-HPLC) (47–50), mass spectrometry (MS) (51–54), enzyme-linked immunosorbent assay (ELISA) (55–61), and Western blot analysis (62, 63). The most used assays to detect gluten in food are based on immunological tests (such as ELISA kits) or proteomic approaches involving MS. However, none of them is considered universally adequate for the complete detection of gluten in food matrices. All technologies present advantages and disadvantages depending on specific tasks (Figure 1 and Table 1).

**TABLE 1:** Strengths and limitations of common analytical methods for the detection of gluten proteins/peptides.

Methods	Strengths	Limitations	References
***ELISA***	• Commercially available. • User friendly. • Rapid analysis. • Quantitative analysis of intact gluten.	• Cross-reactivity of antibodies. • Lack of unique kit procedures (different buffers, concentration of antibodies, extraction protocols, etc.). • Lack of certified reference materials. • Over- or under-estimation of the gluten content.	(55–61)
***Western-blot***	• Identification of more reactive proteins or peptides. • Support the ELISA test.	• Less sensitive compared to ELISA assay. • Problems relating to protein quantification.	(62, 63)
***RP-HPLC***	• Protein polymorphism of gluten. • Evaluation of gliadin/glutenin ratio.	• Need to couple to mass spectrometry for protein/peptide identification.	(47–50)
***Electrophoresis***	• Detect small variations in protein size. • Qualitative characterization of proteins/peptides.	• Difficult to separate gluten proteins having similar molecular weights. • Measure of molecular weights are not accurate. • Need to proteomic based analysis for protein sequencing.	(43–46)
***Mass spectrometry***	• Highly sensitive. • Accurate detection of proteins/peptides. • Quantitative analysis.	• Require experienced staff. • Expensive equipment. • Need certified reference materials for accurate quantitation. • Incomplete available databases.	(51–54)

#### 2.2.1. Immunological method: ELISA assay

Immunological methods for gluten protein detection are the most widespread methodologies available so far. ELISAs are the most frequently used by food manufacturers or control authorities to assess the gluten content in food products. Several ELISA kits are available in the market; however, for some of them, the composition of extraction buffers, the nature of the antibodies, and the calibration material are not declared. Furthermore, some kits are specific for detecting wheat gluten, while others detect also rye and barley prolamins.

The majority of commercially available kits are based on the R5 monoclonal antibody, which is only validated by Codex Alimentarius legislation, while other tests are based on different antibodies, such as the G12 and α-20 monoclonal antibodies. Most of them detect only gliadin proteins, excluding the glutenin fraction. Usually, the gluten content is calculated by multiplying the prolamin content, which represents 50% of total wheat protein by a factor of 2. However, several issues have been reported for ELISA measurements (Table 1) that can result in an overestimation or underestimation of gluten amount since the ratio of prolamin/glutenin depends on the wheat variety and the type of food processing (55–61). Due to this overestimation or underestimation of the gluten content by ELISA, alternative analytical methods are urgently needed (64).

An important issue is the quantitative accuracy of processed foods. Bruins Slot et al. evaluated the accuracy and reproducibility of 14 ELISA kits for gluten detection in several food matrices with different degrees of complexity (55). The authors showed that there is no single ELISA method that can accurately detect and quantify gluten in all the different matrices. In a recent study, the gluten content resulted was overestimated, up to six times, in breakfast food products containing rye, or underestimated, up to seven times, in barley-containing food, thus representing a serious health risk for people with CD (59). Technological treatment may affect the limit of detection of gluten by ELISA. For instance, the exposure of wheat to microwave technology was claimed as an efficient method to remove the antigenic properties of gluten. This evidence was based on R5 ELISA, which detected the reduction of immunogenicity to over 99% (65) after microwave treatment. However, a separate study showed that the undetectably of gluten by the R5 antibody was actually due to a failure to extract the gluten proteins from the microwave-treated flour. In fact, gluten was found to be insoluble in the extraction buffers because of the microwave heat treatment. Conversely, after enzymatic hydrolysis, gluten peptides were easily extracted and analyzed by G12 ELISA and mass spectrometry analysis, confirming that microwave treatment does not abolish the immunogenicity of gluten proteins (66).

#### 2.2.2. Proteomic methods: MS-based technologies

Mass spectrometry (MS)-based proteomic offers an alternative method to detect the gluten content and/or to confirm ELISA measurements. This method represents a complementary way that can provide specific information, such as peptide identification and relative peptide abundance. Furthermore, the analysis of intact proteins by MS has been used to develop low-resolution fingerprints for grain source identification for the implementation of gluten-free labeling regulations (67). Several approaches for the quantitation of immunogenic gluten peptides by targeted liquid chromatography–tandem mass spectrometry (LC-MS/MS) were published in recent years (68). A bottom-up proteomic approach, which is based on the enzymatic digestion of proteins and identification of the resulting peptides by LC-MS/MS, has been applied to gluten analysis and allowed to select specific peptides to be used as markers for the detection of gluten in complex food matrices. Sealey-Voyksner et al. used the LC-MS/MS method to select six CD-immunogenic wheat gluten peptides in native and processed food samples (51). Similarly, Fiedler et al. identified two wheat marker peptides from α-gliadins, to be used to detect wheat contamination in oats (67). Nine CD-immunogenic peptides from α-gliadins were quantitated by van den Broeck et al. using LC-MS/MS (52). Schalk et al. (64) were the first to establish a link between concentrations of 16 wheat marker peptides and gluten contents using a targeted-quantitative LC-MS/MS method. Gluten contents expressed as the sum of all determined protein types were comparable to those analyzed by RP-HPLC and R5 ELISA in wheat starches with high gluten contents.

More recently, Li et al. identified and quantified gluten peptides from wheat, rye, barley, and oats in different food products (53). Several peptide fragments, including immunotoxic peptides, were found in breakfast cereals, breakfast bars, and powdered drinks, such as the peptide RPLFQLVQGQGIIQPQQPAQLEVIR from γ-gliadin. This sequence comprises a known T-cell epitope, VQGQGIIQPQQPAQL. Another stimulatory peptide, QQPGQGQQPEQGQQPGQGQQGYYPTSPQQPGQGK, derived from the high molecular weight glutenin subunit, was found in both breakfast cereals and bars. Again, the SDQPQQSFPQQPQQK peptide that contained the immunotoxic sequence belonging to γ1-secalin was identified in gliadin/avenin-like seed protein in the breakfast cereals. A comparison to ELISA showed similar results for the wheat-containing products, but discrepancies were noted for the barley-containing products. The authors highlighted the high relevance of mass spectrometry as a reliable tool for the detection of gluten proteins in food and, particularly, for extensive heat-treated foods (53). In conclusion, these studies have proven that proteomics is a useful strategy both for the detection of CD-toxic peptides and the quantification of gluten content in processed food products (53).

#### 2.2.3. The INFOGEST protocol for digestion of gluten proteins in complex food matrices

To date, most of the studies on gluten proteins were conducted without taking into account the role of extensive gastrointestinal digestion, consequently obtaining results with little physiological relevance (21, 22, 26, 35, 69). The gastrointestinal digestion employing the INFOGEST model has recently been applied to study the bioavailability of immunotoxic gluten peptides in complex food matrices (70–73). Mamone et al. (74), investigated the metabolic fate of pasta proteins upon *in vitro* simulated gastrointestinal digestion including an additional step with porcine intestinal BBM hydrolases that mimics peptide degradation at the level of the jejunum. The authors demonstrated that bread and pasta gluten proteins were completely hydrolyzed during *in vitro* digestion. Ogilvie et al. (70) used proteomics and an immunochemical approach to assess the immunogenicity of gluten residues in wheat-based food during simulated *in vitro* digestion. The authors demonstrated that gluten toxic peptides were rapidly released from the food matrix during the intestinal phase.

Recently, Boukid et al. successfully combined the INFOGEST method and target proteomic analysis to detect and quantify gluten peptides relevant to CD pathogenesis (54). The authors demonstrated that 227 peptides were released after gastrointestinal digestion, and nine peptides harbored CD-immunogenic sequences. Interestingly, Olgivie et al. explored the kinetic of immunogenic peptides released from the sourdough bread upon INFOGEST digestion, using a quantitative proteomics analysis and ELISA. The authors demonstrated that although sourdough fermentation affected the degree of gluten hydrolysis, no difference in the immunogenicity profile was shown in the digested product, compared with yeast-fermented bread (73).

## 3. Glutenases: Gluten-degrading enzymes as a new therapeutic frontier in CD

Over the last few years, several strategies have been proposed to detoxify or decrease the immunogenicity of wheat-based food (75). An interesting approach is based on the addition of proteases that are able to hydrolyze immunotoxic sequences. For instance, the use of prolyl endopeptidase (PEP) from *Aspergillus niger* during the brewing process represents a useful method to produce gluten-free barley-based beers (76).

Food-grade protease-based strategy seems efficient in detoxifying moderate quantities of dietary gluten proteins. These glutenases have to degrade gluten immunogenic peptides prevalently in the stomach, thus avoiding any stimulation of the duodenal immune system. Endopeptidases, produced by various plants, bacteria, or fungi, are currently under investigation for their capability to degrade the proline/glutamine-rich gluten peptides in the gastric and upper intestinal tracts (77–82). Shan et al. have shown the capability of bacteria prolyl endopeptidases from *Flavobacterium meningosepticum* (FM-PEP), *Sphingomonas capsulate* (SC-PEP or ALV002), and *Myxococcus xanthus* (MX-PEP) in degrading successfully immunogenic gluten amino acid sequences (78). In detail, the gluten-degrading properties of FM-PEP, SC-PEP, and MX-PEP (stable and active at pH 6–7) were evaluated using two substrates, the 33-mer and smaller sequence, PQPQLPYPQPQLP. The results of this study showed that all PEPs preferentially cleave P-Q bonds that are usually found in immunogenic gluten peptides and that are resistant to gastrointestinal proteases. Edens et al. identified glutenase from the fungus *A. niger* (AN-PEP or ASP) that efficiently hydrolyzed gluten and abolish T-cell stimulatory properties (79). Unlike SC-PEP, FM-PEP, and MX-PEP, which have an optimum pH activity between 7.0 and 8.0, AN-PEP is active at lower pH values between pH 4.0 and 5.0 explaining its functionality in the stomach and allowing early-digestion of gluten proteins.

Again, a mixture of aspergillopepsin from *A. niger* and DPP-IV from *Aspergillus oryzae* was used to hydrolyze small amounts of gluten *in vitro* (80). Other studies showed the proteolytic abilities of a glutamine-specific cysteine endoprotease derived from seeds of germinating barley (EP-B2). Different from PEP, EP-B2 has cleavage-site specificity for post-glutamine residues. Gass et al. proposed a new strategy based on the combination of EP-B2 and PEP enzymes, both with gastric activity and complementary substrate specificity (81). Both *in vitro* and *in vivo* functional experiments demonstrated that these enzymes synergically hydrolyzed gluten proteins, reducing their immunogenicity (81).

Recently, a novel microbial endopeptidase expressed in recombinant actinomycete *S. lividans* TK24, named the endoprotease-40 (E40), has been demonstrated to be a successful enzyme in detoxifying gluten proteins during the transit in the stomach, suggesting its use for oral enzymatic therapy (OET) in CD. The authors, using both proteomic and immunochemical approaches (SDS-PAGE, RP-HPLC, LC-MS/MS, and ELISA), showed that E40 efficiently hydrolyzed the most immunogenic 33-mer, as well as the whole gliadin proteins. Furthermore, the analysis of T lymphocytes from duodenal biopsies of patients with CD indicated a markedly reduced production of IFN-γ when exposed to gluten digested with E40, as described below (77, 82).

## 4. Functional approaches to assess the immunostimulatory properties of gluten peptides: Celiac T-cell-based bioassays

As mentioned previously, the peculiar amino acid composition of wheat gluten proteins, and homologous proteins of rye and barley, strongly hampers the degradation by gastrointestinal proteases, with a release of large peptides with immunogenic potential for T cells in the gut lumen of subjects with CD (24, 35, 83, 84). These partially digested gluten peptides are deamidated by the tTG enzyme highly expressed in the intestinal mucosa of patients with CD. This reaction increases the binding affinity of gluten peptides to the CD-associated HLA-DQ2/DQ8 molecules. The peptide-HLA complex is recognized by CD4 + T cells triggering an inflammatory reaction that leads to a profound remodeling of the intestinal mucosa tissue (24, 35, 83, 84). Of note, the gluten-reactive CD4 + T cells are exclusively found in the gut mucosa of patients with CD and persist in the celiac intestinal mucosa for decades as memory cells (85, 86).

For their high disease specificity, these memory T cells reactive to gluten peptides have been largely used in several studies aimed to elucidate the CD immunopathogenic mechanisms and to validate gluten detoxifying and immunomodulatory strategies. Through painstaking work, gluten-specific T cells can be isolated from gut mucosa biopsies of patients with CD and *in vitro* expanded as polyclonal CD3 + cell lines or cell clones, as reported in detail elsewhere (87–90). In brief, intestinal cells are isolated from jejunal explants by enzymatic digestion with collagenase and *in vitro* stimulated with autologous feeder cells and an enzymatic digest of gluten/gliadin proteins. The T-cell cultures are further expanded by repeated stimulations with allogenic feeders, the mitogen phytohemagglutinin, and growth factors IL-2 and IL-15. Usually, the T-cell line antigen specificity is assessed by the detection of IFN-γ, the dominant cytokine in CD pathogenesis, by enzyme-linked immunosorbent assay (ELISA or ELISPOT) and T-cell proliferation assays, as reported in the many studies mentioned earlier. Examples of studies using intestinal T-cell assays in CD are presented in Table 2.

**TABLE 2:** *In vitro* T-cell-based assay to evaluate immunogenicity of gluten proteins and novel strategies to treat celiac disease (CD).

Purpose of the research	Outcomes by *in vitro* functional T-cell assays	References
***Elucidation of immunopathogenic mechanisms*** ***of celiac disease***	Generation and expansion of gliadin-specific T-cells and clones from celiac small intestinal mucosa.	(88–90)
	Characterization of tissue-resident memory T-cells in celiac disease.	(85, 86)
	Identification of immunogenic gluten peptides.	(23, 25, 35, 84)
***Efficacy evaluation of novel enzymatic strategies to treat celiac disease in pre-clinical studies***	Gluten peptide transamidation (mTG-transamidation) of wheat flour inhibits the response to gliadin.	(87, 96)
	Gluten proteolysis at gastric condition by the endoproteases glutenases”: (1) EP-B2/PEP (ALV003/Latiglutenase in clinical trial) (2) Kuma062 (3) E40.	(77, 81, 82, 91, 92)
***Searching of less immunogenic wheat species for CD prevention***	Assessment of the lower immunostimulatory activity of *Triticum monococcum*.	(22, 97, 98)

### 4.1. Identification of gluten immunogenic peptides

For many years, studies aiming to identify the complete repertoire of gluten epitopes were supported by the use of intestinal T-cell lines and T-cell clones generated from the jejunal mucosa of patients with CD (25, 35). These studies demonstrated a great heterogeneity of immunogenic gluten peptides. T-cell epitopes were identified in all wheat gluten proteins (α-, γ-, ω-gliadins, high molecular weight-HMW, and low molecular weight-LMW glutenins) and in homologous proteins from hordeins and secalins (35). An update of the known HLA-DQ-restricted gluten T-cell epitopes, responsible for the induction and/or maintenance of intestinal mucosa inflammation in patients with CD, has been reported (23). The definition of the complete repertoire of gluten immunogenic peptides is a pivotal step for the development of alternative strategies to the gluten-free diet (GFD) for the treatment of CD, as it provides a useful tool to assess whether these novel strategies under investigation succeed in reducing the stimulatory capability of gluten epitopes known as the most dominant in patients with CD.

### 4.2. Analysis of glutenase capability to detoxify gluten peptides

Before moving on to *in vivo* clinical studies and subsequent clinical trials, the efficacy of potential detoxification strategies of gluten proteins from different foodstuff sources has been often assessed using different gluten-reactive T cell lines (G-TCLs) characterized by a large repertoire of reactivity toward gluten peptides (25). Among the several alternative strategies under investigation for CD treatment, great attention is currently paid to enzymatic approaches intended to fastly digest gluten proteins that work at the same low pH occurring in the stomach. As extensively described earlier, many glutenases, produced by bacteria, fungi, and plants, as barley, or engineered recombinant proteins, have been investigated as possible drug therapy for CD alternative to GFD, or as oral supplements to support GFD and protect celiac diseases from unintentional gluten exposures (77, 81, 91, 92). Glutenases, thanks to their capability in cleaving the proline-rich and glutamine-rich gluten sequences, represent a promising drug for oral therapy in CD (93). Pioneering studies from Khosla et al. assayed a combination of two proline-specific endopeptidase (EP-B2/PEP) that operates under gastric conditions is able to detoxify gluten within 10 min of simulated duodenal conditions, as proved by chromatographic analysis and *in vitro* celiac T cells. In particular, they observed that polyclonal G-TCLs, reactive to a panel of known gluten epitopes, did not proliferate in response to all tested concentrations of digested and enzyme-treated gluten (81, 91). Following these encouraging *in vitro* findings, clinical studies have been performed to assess the efficacy of EP-B2 combined with an endopeptidase from *Sphingomonas capsulata* (SC-PEP) known as ALV003/Latiglutenase (94, 95).

Wolf et al. reported *in vitro* celiac T-cell studies to assess the glutenasic activity of a computationally designed kumamolisin endopeptidase known as Kuma030 or TAK062 (92). This recombinant protein recognizes tripeptide sequences in the immunogenic regions of gliadin or homologous proteins in barley and rye. A decreased immunostimulatory potential of gliadin proteins treated with Kuma030 was measured in G-TCLs generated from five patients with CD, as shown by IFN-γ and cell proliferation readouts (92).

**FIGURE 2:**
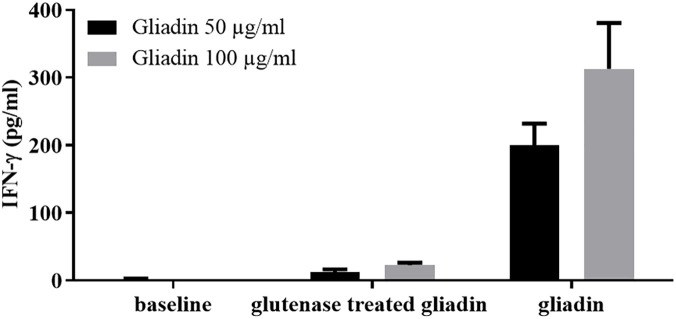
The digestion with the endoproteases “glutenases” strongly reduced the immunostimulatory capacity of gliadin on celiac intestinal T cells. INF-γ responses of intestinal T-cell line obtained from a celiac patient were evaluated after incubation of gliadin proteins both native or treated with glutenase E40 (77, 82). Gliadin was purified from hexaploid wheat flour and incubated with E40 at pH 4, 37°C for 120 min (enzyme:substrate ratio 1:20). Gliadin enzymatic digest was next deamidated by tTG treatment. IFN-γ production was measured by ELISA in cell supernatants collected after 48 hours of incubation. Native and E40-treated gliadin were assayed on T cells at 50 and 100 μg/ml.

Intestinal T-cell lines were also used in a functional assay to verify the reduced immunostimulatory capacity of gluten proteins digested with the E40, a promising protease of microbial origin (77) (Figure 2). A recent study from the same research group showed that E40 was efficient in hydrolyzing gluten epitopes in complex food matrices, such as pasta, bread, and wheat beer samples (82).

### 4.3. Validation of gluten detoxifying strategies aimed to obtain gluten-free wheat flour

An enzymatic strategy to reduce gluten load consists of wheat flour pre-treatment with microbial transglutaminase (mTG) and acyl-acceptor molecules (L-lysine, glycine ethyl ester, and hydroxylamine). This approach consists of inducing the transamidation of specific glutamine residues, thus blocking their deamidation by tTG (87, 96). As the deamidation is a crucial step for the immunotoxicity of gluten proteins, it has been demonstrated that this biochemical strategy strongly reduces the capability of transamidated gluten to stimulate G-TCLs obtained from 12 patients with CD and highly reactive to untreated gliadin (87). The transamidation reaction specifically masks the gluten epitopes rendering them not able to activate pro-inflammatory T cells. Of note, this approach does not alter the integrity of the gluten protein structure, as well as its viscoelasticity and technological properties.

### 4.4. Searching for safer wheat species for CD prevention

**FIGURE 3:**
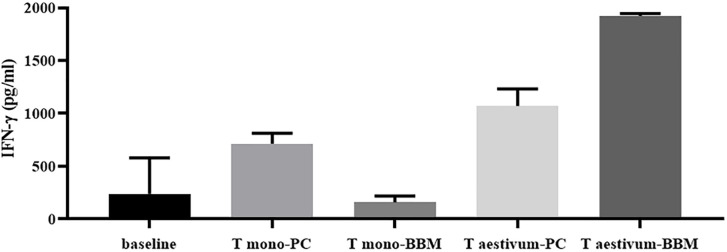
Marked reduction of specific T-cell response to gliadin from diploid wheat (*monococcum*) after the extensive *in vitro* gastro-intestinal digestion including brush-border membrane enzymes (BBM). IFN-γ responses of intestinal T-cell line obtained from a celiac patient were evaluated after stimulation with PC- (pepsin-chymotrypsin) or BBM- (pepsin-chymotrypsin-elastase and brush-border membrane enzymes) gliadin digests from *Triticum aestivum* and *Triticum monococcum* wheats (22). Gliadin samples were deamidated by tTG treatment and assayed at 50 μg/ml. IFN-γ production was evaluated by ELISA in cell supernatants after 48 hours of incubation.

Although the minimal level of gluten consumption that is safe for patients with celiac disease has not yet been established, a number of studies demonstrated that the magnitude of inflammatory T-cell response to gluten strictly depends on the gluten peptide concentration that is loaded on HLA-DQ2/8 restriction molecules. As a consequence, great attention was paid to the research of cereal species with a low content of immunotoxic sequences, with the aim of limiting dietary exposure to gluten in the general population with genetic CD risk (11). As extensively described earlier, another crucial factor determining the immunostimulatory potentiality of gluten proteins is the digestibility by gastrointestinal proteases, strictly dependent on primary protein structure. Recent studies reported that diploid wheat species, such as *T. monococcum (T. monococcum)*, contain gluten proteins more susceptible to the digestion of gastro-pancreatic and brush-border membrane (BBM) enzymes if compared to those from hexaploid common wheat *T. aestivum* (97). For this peculiarity, the diploid wheat species have been tested *in vitro* to ascertain their low immunostimulatory activity on TCLs of patients with CD (22, 97). As convincingly demonstrated by functional T-cell assays, the extensive *in vitro* gastrointestinal digestion of monococcum gluten proteins drastically reduced the capability to elicit IFN-γ production and cell proliferation compared to hexaploidy wheat gluten (Figure 3) (97, 98). These *in vitro* studies were further corroborated by *in vivo* short oral challenge as reported below. All these data obtained from functional T-cell assays, combined with proteomic analysis, make diploid wheat crop species among the most suitable candidates for the prevention of CD in people at high risk of developing the disease due to genetic and familiar predisposition.

## 5. Short-term (3 days) oral gluten challenge

The entirety of limitations connected to a lifelong gluten-free diet (GFD), and the low percentage of patients with CD compliant with dietary therapy, mainly during adolescence, prompted the scientific community to search for an alternative treatment for CD (99, 100). Simultaneously with the development of therapeutic strategies, it became necessary to have functional tools to rapidly test their efficacy before moving forward to clinical studies. For decades, the evaluation of gut mucosa damage, in terms of villous eight and crypt depth (Vh/Cd ratio) and intraepithelial lymphocytes (IEL) infiltration, after a long (from 14 to 90 days) gluten consumption has been the gold-standard approach to assessing the validity of novel therapies (101–105). However, this procedure required an endoscopy and is invasive for most patients with CD; furthermore, the long-term gluten exposure, needed to induce intestinal damage, makes this approach too demanding to evaluate the effects of novel therapies.

In addition, the frequent risk of histological evaluation pitfalls and other possible causes, not gluten-dependent inducing mucosa villi atrophy, cannot be excluded. In the early 2,000 s, it developed a minimally invasive strategy to detect gluten-specific T cells in the peripheral blood of GFD-compliant patients with CD who underwent 3 days of clinically controlled gluten consumption, around 6–16 gr gluten/die supplied as bread slides, sandwiches, or cookies (106–109). Basically, gluten primed CD4 + T lymphocytes, usually undetectable (or low detectable) in the peripheral blood of treated patients with CD, are transiently mobilized in the blood and became detectable after 6 days of gluten consumption revealed by sensitive assays, mainly enzyme-linked immunospot (ELISPOT) assay detecting IFN-γ-producing T cells (106–109), and by tetramers technology at flow cytometry or by tetramer assays (110). Over the past few years, the brief oral gluten challenge gave relevant support in the characterization of gluten epitopes active both in adult and pediatric patients with CD (33, 83), as well as allowed the elucidation of cell phenotype and pattern of cytokines produced by gluten-reactive T cells (90, 107, 108, 111, 112).

### 5.1. Short-term gluten challenge, as a pre-clinical evaluation assay of novel gluten detoxification and therapeutic strategies

A number of studies showed that the short-term gluten challenge is a sensitive tool for the pre-clinical assessment of new strategies to detoxify gluten proteins or therapeutic drugs. Tye Din et al. used this non-invasive short-term procedure to validate the efficacy of the combined cysteine endoprotease and prolyl endopeptidase (ALV003/Latiglutenase) (113). An *in vivo* study was conducted on 20 patients with CD on GFD, randomized to consume foods made with gluten (16 g/day) pre-treated with ALV003, or gluten pre-treated with a placebo. The T-cell reactivity against immunodominant α-gliadin epitope 33-mer or whole gliadin, as revealed by IFN-γ ELISPOT, was markedly decreased in volunteers receiving gluten pre-treated with ALV003/Latiglutenase compared to those receiving placebo-treated gluten, thus suggesting the huge potentiality of glutenase degradation to reduce the gluten immunotoxicity (113).

The relevance of the brief gluten challenge to monitor the efficacy of gluten detoxification strategies was further addressed by an Italian study aimed to assess whether the pre-digestion of wheat flour with selected lactobacilli and fungal proteases (hydrolyzed wheat gluten) might decrease, or even abolish, the T-cell immunogenicity in patients with CD (114, 115). Twenty subjects with celiac disease including pediatric subjects, following a GFD regimen for at least 3 years, were randomized to consume bread slices produced with hydrolyzed wheat flour (corresponding to 10 g of gluten/day). The hydrolyzed wheat gluten did not mobilize gluten-reactive T lymphocytes from the intestinal mucosa to the peripheral blood compared with that elicited after a short-term oral challenge with untreated wheat flour, with statistically significant differences in the number of circulating gluten-reactive T cells between the two experimental groups. Notably, the brief challenge of the study was a further confirmation of a previous long-term (60 days) challenge addressed to demonstrate whether the enzymatic pre-digestion with lactobacilli and fungal proteases abolish gluten protein toxicity (114). No alteration was reported as histological mucosal change or in antibody seroconversion, thus indicating no relapse after pre-hydrolyzed wheat flour long consumption.

**FIGURE 4:**
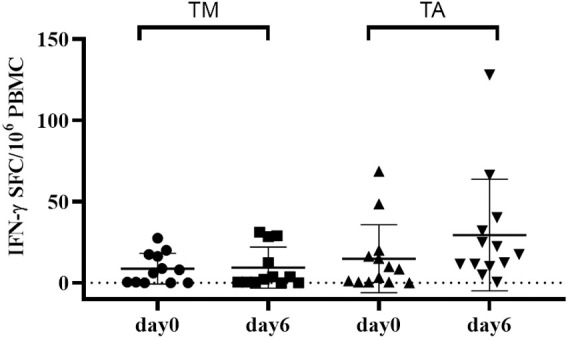
Short gluten oral challenge with the diploid *Triticum monococcum* wheat results in a lower immune response compared to soft *Triticum aestivum* wheat. Gliadin-specific T-cell responses were elicited by the short oral challenge with the diploid wheat *Triticum monococcum* (TM, left panel) and with the hexaploid wheat *Triticum aestivum* (TA, right panel). Celiac subjects (*n* = 11) consumed for 3 days sandwiches made with TM (cv Norberto-ID331) or with TA (cv Sagittario) corresponding to 12 g of gluten (119). PBMCs obtained soon before (day 0) and (day 6) after the gluten consumption were in vitro stimulated with deamidated, pepsin-chymotrypsin digests of gliadin from either TM wheat (left) or TA wheat (right). Digested samples were deamidated by tTG treatment and assayed at 50 μg/ml. IFN-γ secreting cells were revealed by EliSpot assay. Data are reported as IFN-γ spot-forming cells (SFC) detected in 10^6^ PBMCs.

Another successful application of a brief gluten oral challenge was provided by a study aimed to assess the immune stimulatory properties of gluten derived from a diploid wheat species, that is, *T. monococcum.* After the promising *in vitro* T-cell findings (98, 116–118) and proteomic and mass spectrometry analysis demonstrating the pronounced digestibility of diploid gluten proteins (22) compared to the more evolved wheat genome, subsequently a brief oral gluten challenge study was performed (119). Volunteers with CD were enrolled to consume bread sandwiches made with diploid or hexaploid wheat flour (approximately 12 g of gluten per day for each study branch). The results confirmed as gluten from *T. monococcum* retains a reduced capability to recruit *in vivo* gluten-specific T-cell response compared to *T. aestivum* (soft) wheat. A significantly decreased number of IFN-γ secreting cells reactive to diploid gliadins were elicited in the peripheral blood of patients with CD eating sandwiches made with *T. monococcum* flour. Furthermore, the consumption of monococcum-based bread for a few days elicited a lower number of T cells reacting to five immune-dominant epitopes from α-, ω-, and γ-gliadins compared to those elicited by soft wheat. Overall, these findings confirmed the reduced *in vivo* immunogenicity in patients with CD of diploid *T. monococcum* compared to hexaploidy soft wheat (Figure 4). Additional studies in a larger celiac cohort, or in CD first-degree relatives, are required to address the applicability of this grain, with low immunogenic gluten, in the prevention of celiac disease in the general population (119).

Based on that, the short gluten challenge proved to be a reproducible assay to monitor the immune response to gluten, with great potential for its application in clinical practice. The design of clinical trials aimed to evaluate novel therapeutic drugs, or alternative cereals, could benefit greatly from this non-invasive short-term procedure. Examples of studies using short oral gluten challenges in CD are presented in Table 3.

**TABLE 3:** Short oral gluten challenge to evaluate immunogenicity of gluten proteins and novel strategies to treat celiac disease (CD).

Purpose of the research	Outcomes by short gluten oral challenge	References
***Elucidation of immunopathogenic mechanisms of celiac disease***	Detection of gluten-specific T cells in peripheral blood by ELISPOT and tetramers technology.	(106–108, 110–112)
	Identification of immunogenic gluten peptides.	(33, 107)
***Efficacy evaluation of novel enzymatic strategies to treat celiac disease*** ***in pre-clinical studies***	Foods made with gluten pre-treated with glutenase ALV003/Latiglutenase were less immunotoxic for CD patients.	(112, 113)
	Enzymatic pre-digestion with lactobacilli and fungal proteases (hydrolyzed wheat gluten) abolishes gluten proteins toxicity in CD patients.	(114, 115)
***Searching of less immunogenic wheat species for CD prevention***	Reduced immunogenicity in CD patients of diploid *Triticum monococcum* compared to hexaploid *Triticum aestivum* soft wheat.	(119)

## 6. Conclusion

Although nutritionally poor for humans, gluten proteins are largely used in the food industry, as they confer unique viscoelasticity properties to the dough and the palatability of gluten-based food products. Due to the widespread of gluten proteins in foodstuffs, compliance with gluten exclusion dietary therapy might be difficult for many patients with CD. To overcome this, several strategies are currently under investigation that are aimed at detoxifying gluten immunogenic peptides or finding specific therapeutic drugs that aim to recover immune tolerance to gluten.

This review overviewed the analytical and functional methods currently used to detect gluten immunogenic peptides in foodstuffs made with naturally gluten-free cereals, or gluten-containing cereals treated with various detoxifying strategies. The amount and amino acidic sequence of gluten immunotoxic peptides have been characterized in several studies by proteomic and immunochemical analysis, such as liquid chromatography–high-resolution mass spectrometry (LC-MS/MS) and R5/G12 competitive ELISA after the INFOGEST digestive *in vitro* model that mimics the human physiologic gastrointestinal digestion. The capability of digested gluten peptides to stimulate an inflammatory reaction has been dissected both *in vitro*, on intestinal human T cells, and *in vivo*, on peripheral blood of volunteers with CD after a brief oral gluten challenge. The large and successful application of such analytical and functional approaches in pre-clinical studies, aimed to validate biochemical, enzymatic, and immunotherapeutic strategies currently under investigation to treat celiac disease, has been also discussed.

## Author contributions

GM and CG conceptualized the review contents. GM, LD, SV, SP, and CG revised the literature and wrote the manuscript. All authors approved the final manuscript.

## Footnotes

1
http://faostat.fao.org/
